# Diagnostic accuracy of Alvarado scoring system relative to histopathological diagnosis for acute appendicitis: A retrospective cohort study

**DOI:** 10.1016/j.amsu.2022.104561

**Published:** 2022-09-02

**Authors:** Muhammad Tayyab Naeem, Muhammad Amir Jamil, Muhammad Imran Anwar, Hassan Raza, Ali Asad, Hashaam Jamil, Muhammad Junaid Tahir, Jharna Bai, Tabssum Mohamad Ejaz Chauhan, Muhammad Sohaib Asghar

**Affiliations:** a Shaikh Zayed Hospital, Lahore, Pakistan; b Services Hospital, Lahore, Pakistan; c Lahore General Hospital, Lahore, 54000, Pakistan; d Shaheed Mohtarma Benazir Bhutto Medical University (SMBBMU), Larkana, Pakistan; e Tver State Medical University, Tver, Russia; f Dow University of Health Sciences–Ojha Campus, Karachi, Pakistan

**Keywords:** Appendix, Pathology, Diagnosis, Surgery, Sensitivity, Specificity

## Abstract

**Background:**

Acute appendicitis (AA) is a surgical emergency that requires prompt diagnosis and suitable management. It may lead to complications resulting in mortality. To evaluate the diagnostic accuracy of the Alvarado scoring system (ASS) for acute appendicitis concerning histopathological data.

**Methodology:**

About 120 patients were selected for this study consisting of 96 males and 24 females age between 20 and 60. Alvarado scoring system is calculated for each patient after collecting data about demographics, laboratory findings, and clinical examination. Then, we compared it with histopathological diagnosis taking it as a gold standard. Sensitivity, specificity, positive predictive value (PPV), and negative predictive value (NPV) were calculated. SPSS version 20 was used for analyzing the data.

**Results:**

About 120 patients were included in our study. The male to female ratio was 3:1. Sensitivity and specificity were 83.3% and 41% respectively. While PPV and NPV were 85% and 41% respectively. The negative appendectomy rate was 21%. The area under the curve for receiving operating characteristics is 0.628.

**Conclusion:**

ASS is a useful diagnostic tool regarding sensitivity and positive predictive value, especially in developing countries. It is cheap, reliable, and can be easily applied.

## Highlights

• We evaluated the diagnostic accuracy of the Alvarado scoring system (ASS) for acute appendicitis.
• Sensitivity and specificity were 83.3% and 41%.
• While PPV and NPV were 85% and 41% respectively.

## 1 Introduction

Acute appendicitis (AA) is the most prevalent abdominal emergency in both developed and developing countries [1]. With a lifetime risk of 1 in 7 [2], that implies that 6% of individuals suffer an attack during their lifetime. AA requires emergency management. If left untreated, it has a high risk of consequences including perforation, peritonitis, and abscess formation, as well as complications associated with the surgical procedures [3]. The mortality rate for elderly people with perforated appendicitis has been reported between 2.3% and 10% [4]. Various approaches are employed to make diagnoses, but the most practical diagnostic modalities are still routine history and physical examination [5]. Of course, an absolute diagnosis can only be made during the operation and histopathologic study of the specimen [6]. Negative appendectomy rate (NAR) was defined as the proportion of histologically normal appendices in those that underwent appendectomy. A negative appendectomy rate of 20–44% is not uncommon, and many surgeons accept a negative appendectomy rate of up to 30% as inevitable [7]. For this reason, removing a normal appendix places a strain on both individuals and healthcare resources [8]. Various diagnostic modalities have been proposed, including clinical scoring systems, computer programs, ultrasonography(US), computed tomography(CT) scans, magnetic resonance imaging (MRI), and laparoscopy [9] [10]. Imaging techniques are fairly precise [11]. Potentially dangerous ionizing radiation (CT), examiner-dependent efficacy (US), and technique-associated morbidity (diagnostic laparoscopy) are some of the key issues with these diagnostic imaging techniques [12,13]. Many crude approaches to diagnose AA have been established, including serial c-reactive proteins (CRP) levels, white cell count (WCC), and bilirubin as diagnostic markers [14], but these methods are not error-free, leading to unnecessary appendectomies resulting in economic and health concerns. In 1986, Alvarado constructed a 10-point Alvarado scoring system(ASS), also known by the acronym MANTRELS, for the diagnosis of acute appendicitis. It comprises various elements, and each element is given either 1 or 2 scores. Migrating right iliac fossa pain, nausea and vomiting, anorexia, rebound tenderness, elevated temperature, and shift of white blood cells (WBCs) to the left are scored 1, while tenderness and leukocytosis are scored 2 [15,16]. The ASS is simple, effective, and easy to use and provides an accurate tool to rule out appendicitis [17]. Patients with ASS scores 5–7 are likely to have AA and those with a score 7–10 are most likely suffering from AA as depicted in Table 1. Patients who get a score of 7–10 should undergo appendectomy, and patients with a score of 5 or 6 are candidates for a CT scan for the diagnosis [18].

**Table 1:** Risk stratification of patients by utilizing the Alvarado Scoring system.

Alvarado Scoring System	Probability of AA	Included in study or not
<4	Less likely	No
5–7	Likely	Yes
7–10	Most likely	Yes

This study was conducted to analyze the diagnostic accuracy of the ASS relative to histopathological analysis in the prediction of acute appendicitis.

## 2 Materials and methods

This is an observational retrospective study conducted at Shaikh Zayed Hospital, Lahore, Pakistan from July 2020 to February 2022. A total of 120 consecutive patients were included in the study. Sample size was calculated by World Health Organization (W.H.O) sample size calculator. The institutional consent was taken from the review board of Shaikh Zayed Hospital, Lahore, Pakistan and the research protocol was registered with the local registry {Unique identification number (UIN): SZMC/IRB/222/2022}.

The inclusion criteria included age between 20 and 60 years, patients with signs and symptoms of AA on clinical evaluation, ASS greater than 4, and no previous appendectomy. Patients with an age less than 20 and more than 60, having appendicular mass or abscess, ASS less than 4, and a previous appendectomy were excluded from the study. Appendicitis is more common in the pediatric age group, but they are excluded from this study because ASS has poor reliability in children as it overestimates the probability of acute appendicitis in children.

The questionnaire comprising of patient's age, gender, other demographics, and elements of ASS was designed to collect the data. The questionnaire was filled by the medical officers attending patients after informed consent. Any information breaching the privacy of the patient was deferred. ASS is comprised of eight components, including migrating pain, anorexia, nausea/vomiting, right lower quadrant, rebound tenderness, the elevation of temperature above 37.3 Celsius, and laboratory findings (leukocytosis and shift to the left). Each has a predictive role in diagnosis and carries a 1 or 2 score depending on the diagnostic accuracy. Each resected specimen undergoes histopathological analysis, and the results were divided into three classes. Group A (Normal Appendix), Group B (Neutrophilic infiltration in the mucosa, submucosa, and muscular along with vascular congestion), and Group C (Complicated Appendix). Only Group B is included in the study. Patients who underwent open appendectomies with Grid Iron incision were included in our study rather than those operated laparoscopically.

Data analysis was done by SPSS version 20 (IBM). The STROCSS 2021 guidelines were adhered to report the study findings [19]. P-value less than 0.05 was considered significant. Pearson's correlation was applied to analyze the association between ASS and histopathological diagnosis. The receiver operating characteristics (ROC) curve was generated to simulate sensitivity, specificity, positive predictive value (PPV), negative predictive value (NPV), and likelihood ratio (LR). The following formulas were applied to calculations:

**Sensitivity** = True Positive/(True Positive + False Negative).

**Specificity =** True Negative/(True Negative + False Positive).

**PPV =** True Positive/(True Positive + False Positive).

**NPV =** True Negative/(False Negative + True Negative).

**LR =** Sensitivity/(100-Specificity).

## 3 Results

**Fig. 1:**
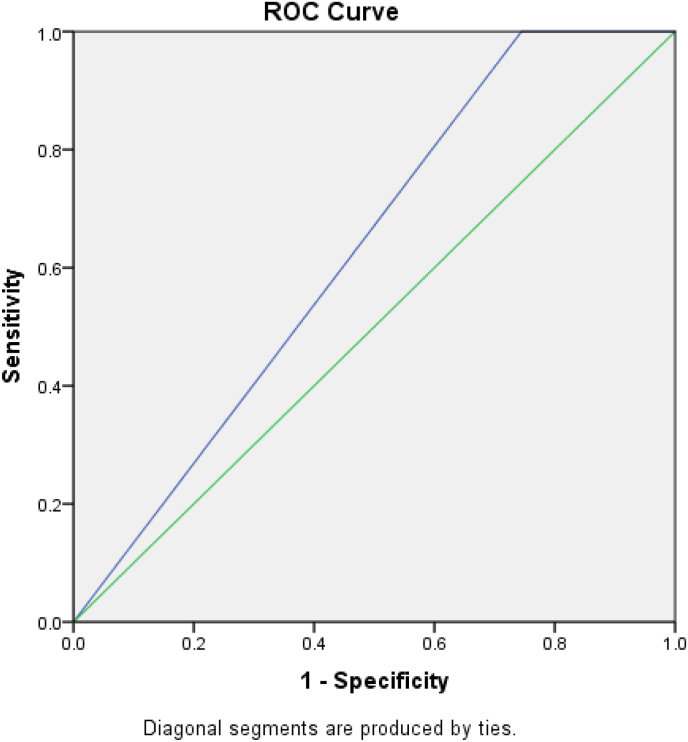
Receiver Operating Characteristics curve (AUC = 0.628 with. CI 95%).

A total of 120 participants were included in the study. Male (n = 92, 76.7%) to female (n = 28, 23.3%) ratio was 3:1. The median age was 31.44 ± 7.875. The major elements of ASS presented are migrating pain (95%), leukocytosis (89%), and rebound tenderness (88%) (Table 2). Patients having an ASS 5–7 were 20%, and 7–10 were 80%. Samples positive for histopathology were 78.33% and those negative for histopathology were 21.66% (Table 3). Sensitivity and specificity for ASS at the cut-off value of 7 were 83% and 41%, respectively. PPV and NPV were 85% and 41%, respectively. Negative appendectomy ASS [[5], [6], [7]] was 41% and for ASS above 7 was 16% with a cut-off value at 7. Pearson correlation was calculated (r = 0.951, p < 0.01). ROC is taken by SPSS with area under curve (AUC) is 0.628(Fig. 1). ROC curve analysis demonstrated more chances of acute appendicitis by increasing of Alvarado score (p = 0.01). The likelihood ratio is calculated to visualize the probability of having AA with a score above 7 and is2.53 in this study.

**Table 2:** Presenting signs and symptoms.

Presenting signs and symptoms	Score	Percentage of each element in present study
Right Iliac Fossa Pain	1	95%
Nausea and Vomiting	1	28%
Anorexia	1	32%
Tenderness	2	47%
Rebound tenderness	1	88%
Elevated temperature (37.3 Celsius)	1	75%
Leukocytosis	2	89%
Shift of white blood cells count to the left	1	51%
**Total**	**10**

**Table 3:** Alvarado Score vs Histopathological Analysis.

Histopathological Analysis	Alvarado Score	
	5–7	7–10
**Yes**	14	80
**No**	10	16

## 4 Discussion

Early diagnosis of acute appendicitis (AA) is required to reduce the morbidity associated with delayed diagnosis. This study showed that ASS has a high diagnostic value in the early diagnosis of AA with high sensitivity (83%). Those having an ASS above 7 have a low NAR (16%), and those having an ASS in the range of 5–7 have a high NAR (41%). Diagnosing AA is an ongoing challenge for most surgeons, because AA presents with atypical symptoms in 50% of the cases [20]. The role of diagnostic imaging, such as US, CT, or MRI, is another major point of contention [21,22]. The application of radiological modalities for the diagnosis of AA is not cost-effective in developing countries, so clinical parameters remain the cornerstone of diagnosis. The clinical scoring system was implemented to make the diagnostic process more straightforward and to refer the patient to an appropriate management plan. Despite medical progress and advancements in diagnostic techniques, approximately 18.2% of appendicitis cases are misdiagnosed [23]. Avoiding appendectomy may lead to perforation, peritonitis, and sepsis, whereas attempting negative appendectomies may result in further morbidity. Various scoring systems were devised to ease the decision regarding AA like Madan, Eskelimen, DeDombal, Ohmann, and Alvarado [24,25], but ASS scoring is most widely used. Stephens et al. have claimed that US is unable to detect AA in 13% of cases, while ASS fails to diagnose 12% cases [26]. Hence US has no benefit over the Alvarado score in the diagnosis of AA.

Our study showed that pain in right iliac fossa is most common symptom of appendicitis in about 95% population, which is compatible with findings concluded in other studies [27,28]. Incidences of leukocytosis and pyrexia are 89% and 75%, respectively. Vigilantly analyzing the patients having a history of AA regarding leukocytosis and pyrexia may reduce the chances of NAR as suggested by Rafiq et al. [29]. Patients with ASS between 7 and 10 should proceed for surgery on an emergency basis because of the increasing probability of inflamed appendix on histology, while those with a score between 5 and 7 should be evaluated further by US, CT scan, and clinic-pathological methods before undergoing surgery because it carries a higher probability of negative appendectomy.

Samples positive for histopathology were 78.33%, which is quite close to a study conducted in Pakistan [30] and UK [31], 84% and 88%, respectively. The permissible rate of negative appendectomy (NA)is about 20% [32,33]. In our study, NAR is 21%, other studies have also concluded the same results as calculated in the present study [1,34]. In Pakistan, three studies [30,35,36] showed that NAR is 15.6%, 17% and 18%, which are also consistent with our findings. Patients having score in the range of 5–7 carry NAR of 41%, while those having score above 7 carry NAR of 16.7% as per our study, so a patient with low ASS will have more chance of negative appendectomy. NA is linked to a high level of morbidity and mortality, including a significant increase in length of stay (LOS), postoperative infection problems, and even death [37]. However, by accepting a larger risk of negative appendectomy, one can effectively buy a reduced rate of perforation [38].

The diagnostic accuracy of any test is determined by its sensitivity, specificity, PPV, and NPV. In our study, sensitivity for ASS at the cut-off value of 7 was 83%, which is comparable with other studies, reporting sensitivity values of 88% [39], 87.41% [40],78% [41], and 77% [42]. Our results have claimed that ASS is specific in 41% at the cut-off value of 7, which is inconsistent with the literatures, which reports a specificity value of 90% [28], 86% [35], 82% [43], and 70% [44]. With regards to our findings, ASS lags as a reliable diagnostic tool to decide for appendectomies with a cut-off value of 7 as far as specificity is concerned.

Our study shows a positive predictive value of 85% comparable with literatures report of 97% [45], 90% [46], 89% [47], and 86.9% [48]. This high predictive value of ASS advocates the utilization of ASS with minimal chances of error. In our study, NPV is 41%, which is also comparable with other studies having NPV of 51% [46], 44% [49], 43% [50], and 33% [51].

There are a few limitations of this study. Ideally, the cut-off value should be set in between 6.5 and 7.5. Increasing the cut-off value increases the probability of true positive cases. A total of 120 consecutive patients were included in the study, so a larger sample size would have made this study more reliable. Retrospective study and the absence of conservative management are not the golden points of study. Patients could not be analyzed over a while due to the cross-sectional sort of study.

## 5 Conclusions

In agreement with the literature survey, our study suggested that those having score in the range of 5–7 should be further evaluated because of the high negative appendectomy rate and those having score above 7 should be prepared for an appendectomy on an urgent basis to prevent perforation. ASS is an effective strategy for surgical residents to proceed with surgery at a cut-off value of 7.

## Peer review and provenance

Externally peer reviewed, not commissioned.

## Sources of funding

None.

## Ethical approval

Ethical approval was taken in this study from institutional review board of Shaikh Zayed Hospital, Lahore, Pakistan (Ref no: SZMC/IRB/222/2022).

## Consent

All participants were selected through a retrospective mechanism hence there was a waiver to obtain written informed consent provided by IRB.

## Authors’ contributions

MTN, MAJ, and MIA conceived the idea; MTN, MAJ, HR and M.I.A collected the data; MTN and MSA analyzed and interpreted the data; MTN, HJ, AA, MJT, and MSA did write up of the manuscript; and finally, MSA, JB, TMEC, MJT, and HJ reviewed the manuscript for intellectual content critically. All authors approved the final version of the manuscript.

## Registration of research studies

1. Name of the registry: Shaikh Zayed Hospital, Lahore, Pakistan.
2. Unique Identifying number or registration ID: SZMC/IRB/222/2022.
3. Hyperlink to your specific registration (must be publicly accessible and will be checked):

## Guarantor

Muhammad Sohaib Asghar and Muhammad Junaid Tahir.

## Declaration of competing interest

We declare no conflict of interests.
